# The cGAS-STING pathway in ischemia-reperfusion injury in acute cerebral infarction: a new therapeutic opportunities?

**DOI:** 10.3389/fneur.2024.1471287

**Published:** 2024-12-17

**Authors:** Jun Hu, Mengxiang Tian

**Affiliations:** ^1^Department of Rehabilitation Medicine, The Affiliated Hospital of Yunnan University, Kunming, China; ^2^Department of General Surgery, Xiangya Hospital, Central South University, Changsha, Hunan, China

**Keywords:** cGAS-STING pathway, acute cerebral infarction, ischemia-reperfusion, cGAS-STING inhibitor, application of nanomaterials

## Abstract

The innate immune response is the body's first line of defense against external pathogens and endogenous damage signals. The cGAS-STING pathway is a crucial component of the innate immune response, playing a key role in initiating antiviral and anti-infective immune responses by recognizing cytosolic DNA. Acute cerebral infarction is one of the leading causes of death and disability worldwide, with the primary treatment approach being the restoration of blood flow to ischemic brain tissue. However, reperfusion injury remains a significant challenge during treatment. The overactivation of the cGAS-STING pathway and its association with ischemia-reperfusion injury have been confirmed in numerous studies. This article will systematically elucidate the mechanisms of the cGAS-STING pathway, its role in ischemia-reperfusion injury in acute cerebral infarction, the current research status of cGAS-STING inhibitors, and the application of nanomaterials in this context, evaluating the therapeutic potential of this pathway.

## Graphical Abstract

Targeted inhibition of the overactivated cGAS-STING signaling pathway after acute cerebral infarction can alleviate ischemia-reperfusion injury and reduce brain tissue damage. 
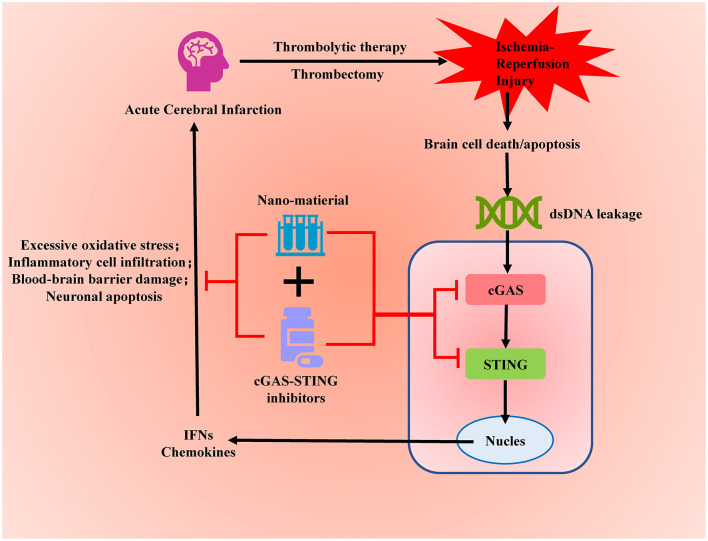

## 1 Backgrounds

In the innate immune system, pathogen-associated molecular patterns (PAMPs) and damage-associated molecular patterns (DAMPs) are crucial signals for recognizing foreign pathogens and host damage. DNA and RNA from bacteria, viruses, fungi, and host cells are important components of these signals (1, 2). When host cells are damaged or undergo necrosis, their DNA can be released into the cytoplasm or extracellular space, becoming DAMPs, and triggering a series of signaling pathways through pattern recognition receptors (PRRs) that activate the innate immune system (3). This enables the host to rapidly identify and respond to bacterial, viral, and fungal infections as well as host cell damage, providing early defense and coordinating subsequent adaptive immune responses. The cGAS (cytosolic DNA sensor) and STING (stimulator of interferon genes) pathway plays a significant role in the innate immune system by detecting abnormal intracellular DNA and initiating an immune response to protect the host from pathogen invasion (4, 5).

Acute cerebral infarction is caused by a sudden interruption of blood supply to the brain. It is one of the leading causes of death worldwide, particularly prevalent and fatal among the elderly (6). This disease poses a significant challenge to global public health. The current international treatment principle for acute cerebral infarction is to restore blood flow to the ischemic brain tissue as quickly as possible (7–9). Although this approach has shown some effectiveness in improving the prognosis of patients with acute cerebral infarction, many challenges remain, with ischemia-reperfusion injury being one of the most critical issues. Ischemia-reperfusion injury (IRI) after acute cerebral infarction refers to the additional damage to brain tissue caused during the process of restoring blood flow (reperfusion) (10). While reperfusion can restore blood supply to the ischemic area and save dying brain cells, it can also trigger a series of complex pathological processes leading to further damage (11–13).

The role of the cGAS-STING pathway in ischemia-reperfusion injury has recently garnered widespread attention. During ischemia-reperfusion, damaged cells release mitochondrial DNA and nuclear DNA fragments into the cytoplasm, activating the cGAS-STING pathway and triggering a strong inflammatory response, which further exacerbates tissue damage (14, 15). Given the critical role of the cGAS-STING pathway in ischemia-reperfusion injury, targeting this pathway may be an effective therapeutic strategy to reduce ischemia-reperfusion injury and improve the prognosis of acute cerebral infarction (16, 17). This article provides a detailed overview of the cGAS-STING signaling pathway's role in ischemia-reperfusion injury in acute cerebral infarction, assesses the potential of this pathway as a therapeutic target, and summarizes the latest developments in cGAS-STING inhibitors and the application of emerging nanomaterials in this field. The aim is to offer a novel and effective treatment strategy to improve the prognosis of acute cerebral infarction.

## 2 Activation mechanism of the cGAS-STING signaling pathway

The cGAS-STING signaling pathway is an important part of the innate immune system. It detects exogenous DNA (such as viral DNA) and endogenous abnormal DNA (such as DNA released from damaged cells) within the cell, activating the production of type I interferons and inflammatory factors to rapidly respond to and inhibit viral infection. Additionally, the cGAS-STING pathway plays a crucial role in tumor immunity and autoimmune diseases (18).

cGAS is a cytosolic DNA sensor that contains a DNA-binding domain and a catalytic domain. Under normal conditions, there should not be any free DNA in the cytoplasm (19). When exogenous or endogenous double-stranded DNA appears in the cytoplasm, the DNA-binding domain of cGAS is responsible for recognizing and binding to the DNA. DNA binding triggers a conformational change in cGAS, including the rearrangement of its active sites, allowing it to catalyze its substrates more effectively (20, 21). After the conformational change, the catalytic domain of cGAS becomes active and can convert ATP and GTP into cyclic GMP-AMP (cGAMP). cGAMP is a cyclic structure formed by a guanosine monophosphate (GMP) and an adenosine monophosphate (AMP) linked by phosphodiester bonds. It has a unique cyclic molecular framework that effectively acts as a second messenger to bind with STING (22).

The STING protein is a crucial signal transduction molecule located on the endoplasmic reticulum (ER) membrane, featuring four transmembrane domains. Its N-terminus is embedded in the ER membrane, anchoring STING firmly to the ER membrane. The C-terminus of STING is situated in the cytoplasm and contains the functional regions necessary for binding with cGAMP and signal transduction (23, 24). When cGAMP binds to STING, it induces a conformational change that activates STING. Activated STING dissociates from the ER membrane and translocates to the Golgi apparatus membrane via a vesicular transport mechanism. The aggregation and oligomerization of STING on the Golgi apparatus membrane are critical steps for activating downstream signaling pathways (25, 26). On the Golgi membrane, STING recruits and activates TANK-binding kinase 1 (TBK1) through its C-terminal domain. Activated TBK1 phosphorylates the C-terminal activation domain (serine/threonine-rich region) of interferon regulatory factor 3 (IRF3) (27). Phosphorylation induces a conformational change in IRF3, transitioning it from an inactive to an active state, allowing it to form homodimers. The dimerization of IRF3 exposes a nuclear localization signal (NLS), which is recognized by nuclear transport proteins, facilitating the translocation of IRF3 into the nucleus. Within the nucleus, dimerized IRF3 binds to specific DNA sequences, initiating the transcription of type I interferon genes (such as IFN-β) and other antiviral genes (28).

Recent studies have found that STING not only activates TBK1 and triggers an antiviral immune response through IRF3 but also activates the NF-κB signaling pathway by activating the IKK complex (29). STING recruits the IKK complex through its C-terminal signaling segment, leading to the activation of the IKK complex with the assistance of STING. The activated IKK complex, particularly IKKβ, phosphorylates serine residues on IκBα (inhibitory κB protein). Under normal conditions, IκBα is bound to NF-κB (such as p65/RelA and p50) in the cytoplasm, inhibiting NF-κB activity. Phosphorylated IκBα is tagged for ubiquitination and subsequently degraded via the 26S proteasome pathway (30). Following IκBα degradation, the NF-κB dimer exposes its nuclear localization signal (NLS), allowing it to be recognized and transported to the nucleus by nuclear transport proteins. Inside the nucleus, the NF-κB dimer binds to specific DNA sequences (κB sites), initiating the transcription of a series of genes and the release of inflammatory chemokines (31).

**Figure 1:**
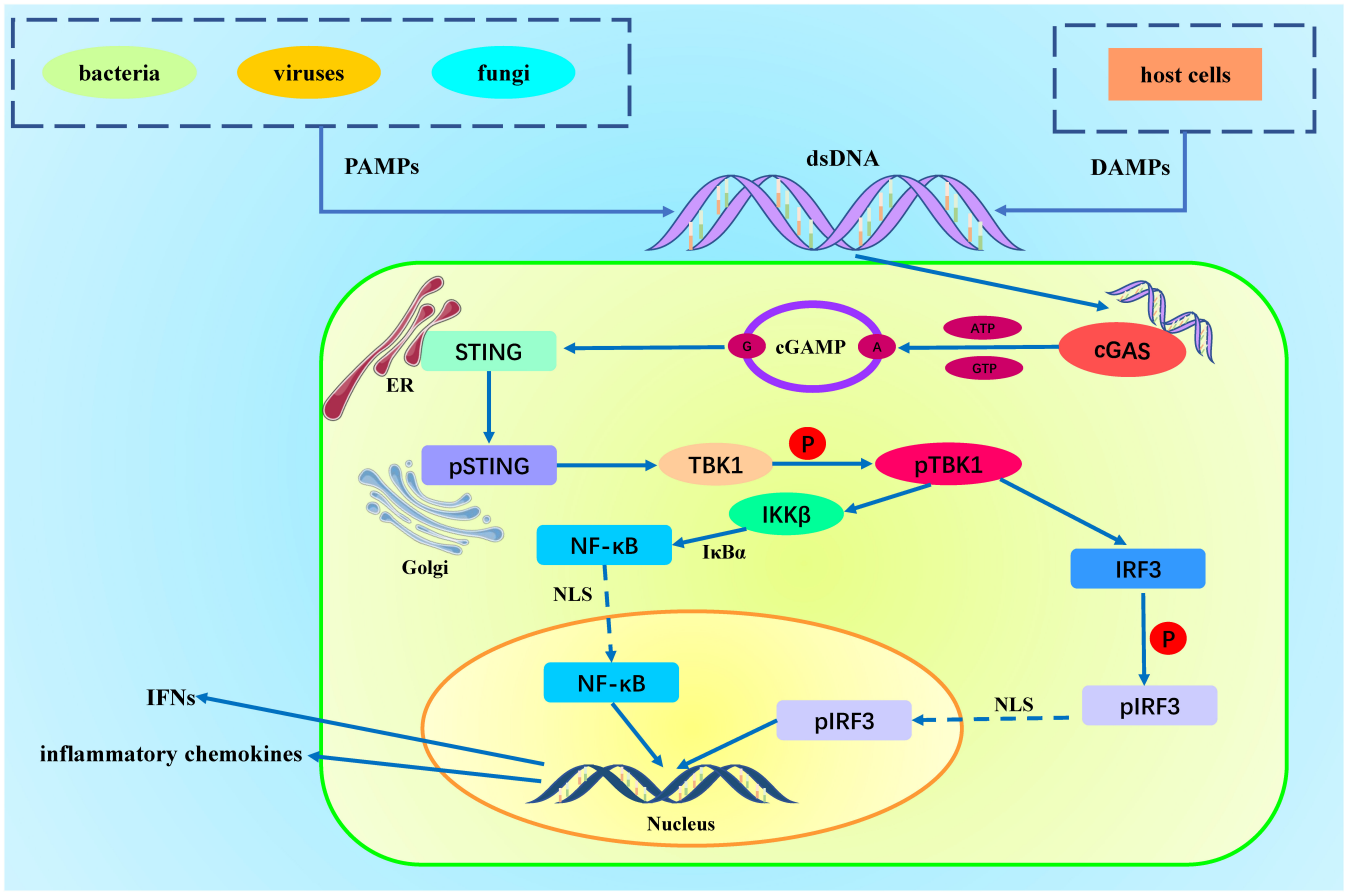
Activation of the cGAS-STING Pathway. DAMPs (Damage-Associated Molecular Patterns) originate from host cell damage or death, such as nuclear DNA and mitochondrial DNA. PAMPs (Pathogen-Associated Molecular Patterns) originate from pathogens, such as viral DNA, bacterial DNA, and double-stranded RNA. Cytosolic DNA is recognized and bound by cGAS, which activates cGAS. The activated cGAS utilizes ATP and GTP as substrates to catalyze the production of cyclic GMP-AMP (cGAMP). cGAMP binds to the STING protein located on the endoplasmic reticulum membrane, inducing a conformational change in STING, leading to its activation. The activated STING protein forms oligomers and translocates to the Golgi apparatus, where it recruits and activates TBK1 (TANK-binding kinase 1). TBK1 then phosphorylates IRF3 (interferon regulatory factor 3). Phosphorylated IRF3 dimerizes and translocates to the nucleus, initiating the gene expression of type I interferons and pro-inflammatory cytokines. Simultaneously, STING recruits the IKK complex (IκB kinase complex) at the Golgi apparatus. The activated IKK complex phosphorylates IκBα (inhibitor of κB), leading to its ubiquitination and degradation, releasing the associated NF-κB dimer (usually p65 and p50). After IκB degradation, the NF-κB dimer (p65/p50) is released and translocates to the nucleus, ultimately promoting the release of chemokines.

In summary, the cGAS-STING signaling pathway plays a critical role in innate immunity by detecting intracellular DNA and initiating downstream IRF3 and NF-κB signaling pathways, triggering robust antiviral and pro-inflammatory immune responses. This pathway is essential not only for combating viral infections but also for DNA damage response and immune surveillance (Figure 1).

## 3 The role of the cGAS-STING signaling pathway in ischemia-reperfusion injury in acute cerebral infarction

Acute cerebral infarction is typically caused by the occlusion of cerebral blood vessels, leading to ischemia and hypoxia in brain tissue. Rapid restoration of cerebral blood flow can save endangered brain cells, reduce irreversible damage, and limit the infarct core area while protecting the surrounding penumbra (the brain tissue that is still alive but functionally impaired), thereby reducing the volume of the infarct (32, 33). Currently, the academic community widely agrees that using methods such as intravenous thrombolysis, intra-arterial thrombolysis, or mechanical thrombectomy to quickly dissolve or remove the thrombus and restore obstructed cerebral blood flow can minimize the ischemic time of brain tissue, help reduce complications such as brain edema and hemorrhage, significantly increase patient survival rates, reduce disability rates, and improve long-term functional outcomes and quality of life (34, 35). However, during the process of restoring blood flow to ischemic tissue, additional damage often occurs due to the rapid restoration of blood flow, known as ischemia-reperfusion injury. This injury can exacerbate the original brain damage and affect the therapeutic effect. The main reasons for reperfusion injury include excessive generation of oxygen free radicals, calcium overload, and excessive infiltration of inflammatory cells (36, 37). To address these issues, experts suggest that measures to counter ischemia-reperfusion injury after recanalization in acute cerebral infarction mainly include antioxidant therapy to reduce oxidative stress damage and calcium channel blockers (such as nimodipine) to reduce intracellular calcium overload. However, treating ischemia-reperfusion injury in acute cerebral infarction remains quite challenging in clinical practice.

Reperfusion injury in acute cerebral infarction involves multiple pathological mechanisms, including the generation of oxygen free radicals, calcium overload, inflammatory response, and disruption of the blood-brain barrier (38). These factors interact with each other, making it difficult to treat the condition by targeting a single cause effectively. However, as research has progressed, scholars have found that excessive generation of oxygen free radicals can attack cell membranes, proteins, and DNA, leading to brain cell damage and death. Similarly, the influx of calcium ions into cells after reperfusion causes intracellular calcium overload, resulting in brain cell death. Significant increases in DNA damage inside and outside cells can be recognized by cGAS, which activates cGAS to produce cGAMP, subsequently activating STING (39). Once STING is activated, it induces the production of large amounts of type I interferons and pro-inflammatory cytokines through the interferon regulatory factor (IRF) and nuclear factor-kappa B (NF-κB) signaling pathways, exacerbating the inflammatory response (40, 41). Excessive activation of the inflammatory response causes a large number of inflammatory cells (such as neutrophils and macrophages) to be activated and accumulate in the ischemic area, releasing various inflammatory mediators that worsen brain tissue damage. Moreover, excessive inflammation may disrupt the blood-brain barrier, increasing the risk of brain edema and hemorrhage, further aggravating brain injury. Therefore, exploring the role of the cGAS-STING signaling pathway in ischemia-reperfusion injury holds promise for providing a new therapeutic approach to overcome the challenges of treating reperfusion injury in acute cerebral infarction (Table 1).

**Table 1:** A summary of the role of cGAS-STING in ischemia-reperfusion injury after acute cerebral infarction.

**Disease**	**Model**	**Method**	**Conclusion**	**Reference**
Ischemic stroke	The mouse model of middle cerebral artery occlusion	Inhibition of microglial M1 polarization using si-cGAS	The cGAS-STING pathway exacerbates cerebral ischemic injury	(42)
		cGAS knockout mice reduce brain tissue damage		(43)
		Administration of the cGAS inhibitor A151 significantly reduced the brain infarct volume in mice		(15, 44, 45)
		DNase I-mediated downregulation of the STING pathway reduces brain hemorrhage		(39)
	The BV2 microglial cell oxygen-glucose deprivation/reperfusion (OGD/R) model	The STING inhibitor C-176 can promote M1/M2 polarization		(46)
Subarachnoid hemorrhage	The mouse intracerebral vessel perforation model of subarachnoid hemorrhage	Evaluation of mouse brain edema and neuronal injury using the STING antagonist C-176 and the STING agonist CMA	Pharmacological inhibition of STING can alleviate inflammation-induced damage in SAH	(47)
Neonatal Hypoxia-Ischemia	The HIE Rice-Vannucci model of rat	siRNA-mediated silencing of STING leads to a reduction in infarct area and improvement in neurological behavior in rats	The cGAS/STING pathway is a potential therapeutic target for inhibiting delayed neuronal death after hypoxia-ischemia	(48)
Cerebral venous sinus thrombosis	The CVST mouse model	The cGAS inhibitor RU.521 and STING siRNA can improve neuronal apoptosis after CVST	Inhibition of the cGAS-STING pathway reduces the neuroinflammatory burden in CVST	(49)

Animal model studies have shown that inhibiting the cGAS-STING pathway can reduce the inflammatory response and tissue damage in ischemia-reperfusion injury of acute cerebral infarction, suggesting the potential of inhibitors targeting this pathway. Previous research has demonstrated that the release of double-stranded DNA from dead brain cells into the cytoplasm in acute cerebral infarction activates the cGAS-STING pathway and induces inflammation. Using siRNA to inhibit cGAS, knocking out microglial cGAS, or silencing STING molecules can alleviate acute cerebral infarction inflammation, reduce cell death, decrease infarct volume, and mitigate neurological deficits (39, 42–44).

Building on this, Li et al.'s research found that mitochondrial DNA-induced activation of the cGAS-STING pathway was observed in the brains of rats with ischemia-reperfusion injury. Knocking out microglial cGAS and inhibiting STING improved neurological function, reduced acute cerebral infarction ischemia-reperfusion injury, inhibited the cascade of neuroinflammation, and rescued many damaged but not dead neurons in the ischemic penumbra following reperfusion injury (45). In mice experiments, the researchers used a middle cerebral artery occlusion (MCAO) model in mice and an oxygen-glucose deprivation/reperfusion (OGD/R) model in BV2 microglial cells to simulate acute ischemic cerebral infarction. They found that the specific STING inhibitor C-176 reduced brain edema and neuronal damage caused by ischemia-reperfusion, eliminating the adverse effects of cGAS-STING overactivation on acute cerebral infarction outcomes (46).

Zhao et al. conducted in-depth studies on ischemia-reperfusion injury and autophagy following acute cerebral infarction. First, they used the selective cGAS inhibitor RU.521 in a mouse model of cerebral ischemia-reperfusion injury, which alleviated oxidative stress, NCOA4-mediated ferritinophagy, apoptosis, and infarct volume associated with CIRI (15, 47). They then applied the cGAS inhibitor RU.521 in an OGD/R model of HT22 cells and found that it effectively inhibited CIRI-induced ferritinophagy, oxidative stress, and cell damage. Zhao et al.'s research validated in both animal and cell models that the activation of the cGAS-STING pathway exacerbates ischemia-reperfusion injury after cerebral infarction by inducing NCOA4-mediated ferritinophagy (48).

**Figure 2:**
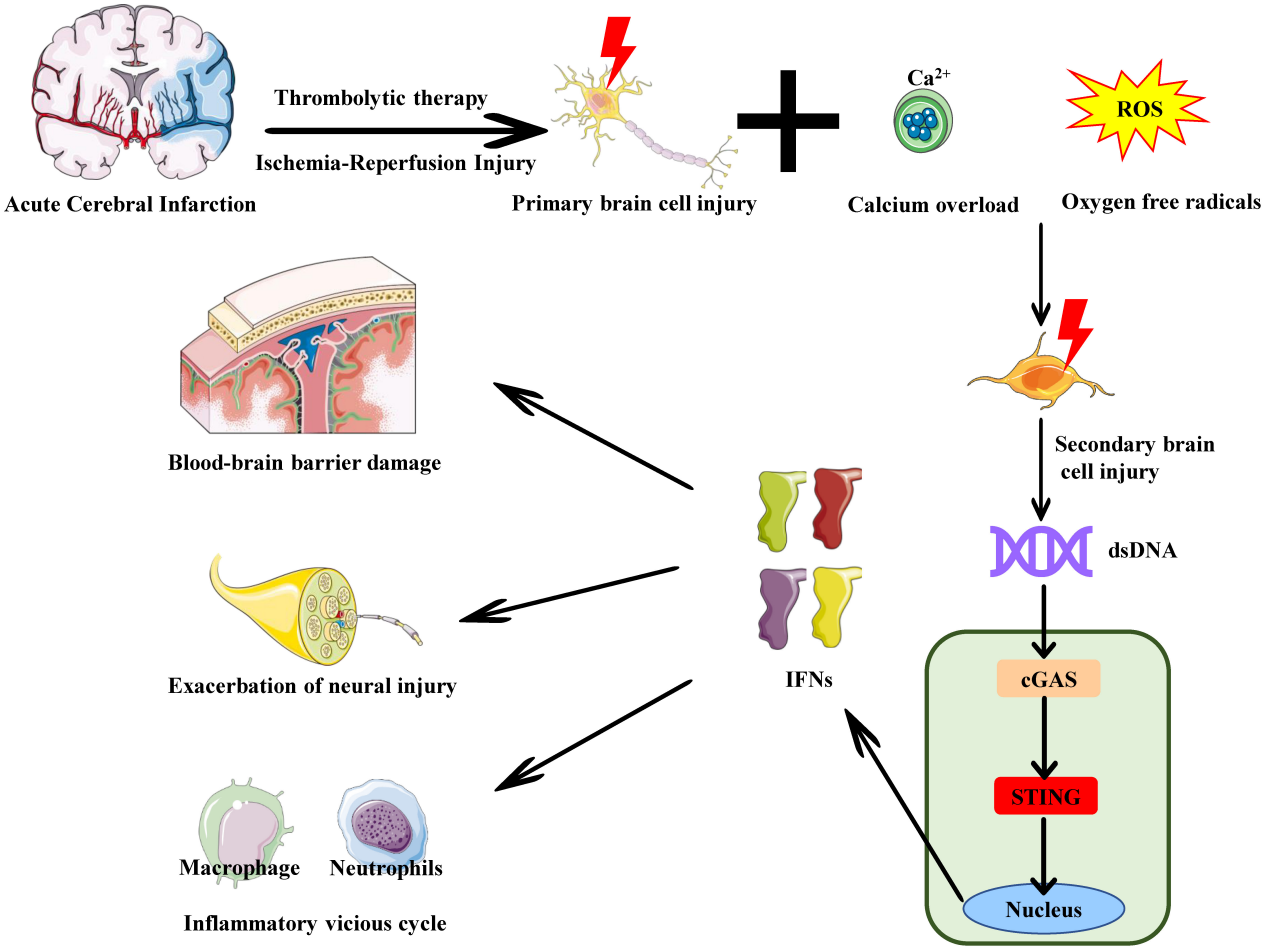
The role of the cGAS-STING pathway in reperfusion injury in acute cerebral infarction. In acute cerebral infarction -induced ischemia-reperfusion injury, neuronal death and rupture release large amounts of dsDNA, such as cytosolic DNA and mitochondrial DNA. This cytosolic DNA is released into the extracellular space or enters the cytoplasm of other cells. cGAS recognizes and binds to this cytosolic DNA from the dsDNA, activating the cGAS-STING pathway and inducing the release of IFNs. The release of these cytokines enhances the local inflammatory response, attracting immune cells (e.g., neutrophils, macrophages) to the site of brain injury. Persistent pro-inflammatory signaling amplifies the inflammatory response, further damaging brain tissue and increasing blood-brain barrier permeability. This leads to the infiltration of more cytokines and inflammatory cells into the brain tissue, expanding the damage area and resulting in neuronal apoptosis and exacerbation of neural injury.

Regrettably, current research on the cGAS-STING pathway and ischemia-reperfusion injury in acute cerebral infarction is mainly focused on animal and cell models. So far, there have been no clinical studies related to the cGAS-STING pathway in acute cerebral infarction -related ischemia-reperfusion injury. However, considering the existing evidence indicating the crucial role of the cGAS-STING pathway in ischemia-reperfusion injury in acute cerebral infarction, future research needs to further explore the specific mechanisms of this pathway and conduct clinical studies to verify the efficacy and safety of therapeutic strategies targeting the cGAS-STING pathway in patients (Figure 2).

## 4 Current status of cGAS-STING inhibitor development

The cGAS-STING signaling pathway has emerged as a promising therapeutic target for various diseases due to its essential role in immune responses and inflammation. Recently, numerous companies and institutions worldwide, including prominent players such as Novartis and GlaxoSmithKline, have made significant advancements in developing inhibitors targeting this pathway. These agents are primarily intended for the treatment of infectious, inflammatory, and autoimmune diseases (Table 2).

**Table 2:** Summary of cGAS -STING pathway inhibitors.

	**Classification**	**Subclassification**	**Name**	**Reference**
cGAS inhibitors	Interfere with the interaction of cGAS with its natural substrates	Non-substrate competitive inhibitors	RU.521	(49)
		Substrate-competitive inhibitors	G150	(50)
			I-a-9c
	Target the catalytic site of cGAS	NA	PF-06928215	(51, 52)
STING inhibitors	Competitive antagonists	NA	H-151	(53–55)
			Astin C	
			SN-011	
			Compound 13	
			Compound 18	
	Antagonists targeting palmitoylation sites	NA	C-176	(56, 57)
			C-178	

cGAS inhibitors can be broadly classified into two categories based on their mechanisms of action: those that interfere with the interaction between cGAS and its natural substrates (ATP or GTP) or product (cGAMP), and those that target the catalytic site of cGAS. Catalytic site inhibitors bind directly to the active site of cGAS, blocking its interaction with natural substrates or hindering its catalytic function. These include both non-substrate competitive inhibitors and substrate competitive inhibitors (20, 49). A representative non-substrate competitive inhibitor, RU.521, binds to cGAS and prevents its activation, effectively inhibiting the cGAS-STING pathway and reducing inflammation in multiple disease models (50). Substrate competitive inhibitors, on the other hand, directly compete with cGAS's natural substrates for binding to the catalytic site, thus inhibiting its enzymatic activity. These inhibitors mainly mimic cGAS's natural substrates and bind competitively to the active site, thereby preventing catalysis. Notable examples include G150 and alternative acylamide compounds, such as I-a-9c (51). I-a-9c inhibits cGAS activity effectively through π-π stacking and π-cation interactions with cGAS's aromatic center, as well as hydrogen bonding with Asp 227 (52). Inhibitors that block cGAS's interaction with its natural substrates or product function by preventing its activation and activity, including substrate competitive inhibitors like PF-06928215, which competes for the catalytic site, blocking ATP and GTP interactions and subsequently inhibiting cGAMP production (53, 54). By various mechanisms, these inhibitors suppress cGAS activity and thereby block downstream STING signaling.

STING inhibitors have also been developed into two main categories: competitive antagonists and antagonists targeting the palmitoylation site (55). Competitive antagonists inhibit STING signaling by occupying its cyclic dinucleotide (CDN) binding site, thus preventing binding of natural ligands, such as cGAMP. Examples include H-151, Astin C, SN-011, Compound 13, and Compound 18 (56–58), among which H-151 has shown significant therapeutic effects in models of inflammatory and autoimmune diseases (59–62). Antagonists targeting the palmitoylation site, including C-176 and C-178, inhibit STING activation by disrupting palmitoylation near its transmembrane region, thereby blocking palmitoylation modification (63, 64). Both categories of STING inhibitors demonstrate potential in modulating immune and inflammatory responses.

In current ischemic stroke-reperfusion injury models, cGAS-STING inhibitors have shown promising efficacy. RU.521, a cGAS inhibitor, has been demonstrated to improve motor function and reduce infarct volume in rat and mouse models of ischemic stroke induced by middle cerebral artery occlusion. It also inhibits neuronal apoptosis and promotes neurotrophic factor expression, suggesting that cGAS inhibition may suppress neuronal apoptosis and inflammation while enhancing the expression of neuroplasticity and angiogenesis-related proteins (15, 65). This effect has been further validated in cellular models (48). Similarly, STING inhibitors exhibit comparable therapeutic potential. Researchers employed the STING-specific antagonist SN-011 in mice with ischemic stroke induced by middle cerebral artery occlusion, resulting in reduced early inflammation and improved stroke pathology through altered phagocyte activity that supports myelin regeneration (66). These findings affirm that targeting the cGAS-STING pathway with inhibitors could mitigate the inflammatory response associated with ischemia-reperfusion injury after stroke, providing a novel therapeutic strategy to enhance post-stroke recovery and reduce neuronal damage.

## 5 Application of nanomaterials in acute cerebral infarction

Research on nanomaterials for targeting the inhibition of the cGAS-STING pathway has shown great potential. By designing and functionalizing nanomaterials, scientists can effectively deliver cGAS-STING inhibitors, enhancing drug stability, extending their circulation time in the body, and improving targeting to enhance therapeutic efficacy. Nanoparticles can serve not only as drug delivery systems but also, through the modification of targeting ligands or functional molecules on their surface, achieve targeted delivery to specific cells or tissues (67). Additionally, multifunctional nanomaterials can deliver cGAS-STING inhibitors and carry other therapeutic molecules (such as anti-inflammatory drugs) to achieve synergistic therapeutic effects (68).

Currently, there have been reports on the use of nanomaterial technology to target the cGAS-STING signaling pathway for treating ischemia-reperfusion injury in acute cerebral infarction. Sun et al. developed an intelligent multifunctional delivery system to target ischemic brain tissue and deliver a peptidylarginine deiminase 4 inhibitor. In a middle cerebral artery occlusion/reperfusion (MCAO) mouse model, this system not only inhibited the release of NETs during ischemic cerebral infarction but also further suppressed the cGAS-STING signaling pathway, significantly reducing ischemia-reperfusion injury, decreasing infarct size, and lowering mortality (69).

Additionally, Zhu et al. designed a DNase-mimicking artificial enzyme loaded with the STING inhibitor C-176 to target ischemic brain tissue in an MCAO mouse model. The nuclease component cleared mtDNA within brain cells, while C-176 directly inhibited downstream STING. This combination synergistically alleviated inflammation caused by the activation of the cGAS-STING innate immune pathway, thus protecting neurons from secondary inflammatory damage after acute cerebral infarction reperfusion (70).

These findings illustrate the significant potential of nanomaterials in targeting and inhibiting the cGAS-STING pathway for treating ischemia-reperfusion injury in acute cerebral infarction. By optimizing the design, surface modification, and functionalization of nanomaterials, the delivery efficiency and therapeutic effect of cGAS-STING inhibitors can be significantly enhanced. Despite some challenges, ongoing research and technological advancements suggest that nanomaterials may play an important role in targeting the cGAS-STING pathway for the treatment of ischemia-reperfusion injury in acute cerebral infarction.

## 6 Discussion

Ischemia-reperfusion injury (IRI) is a significant pathological component of cerebral infarction, where the sudden return of blood flow after ischemic insult paradoxically exacerbates tissue damage through inflammation and oxidative stress. Traditionally, a range of signaling pathways has been studied in the context of IRI, including the toll-like receptor (TLR) pathways (71), the nuclear factor kappa B (NF-κB) pathway (72, 73), and the NOD-like receptor family pyrin domain-containing 3 (NLRP3) inflammasome (74, 75). The cGAS-STING pathway, recently recognized as a crucial mediator of inflammation, adds a novel layer of complexity to our understanding of the mechanisms underlying IRI. The role of cGAS-STING in neuroinflammation, and particularly in ischemic stroke, brings both overlapping and distinct characteristics when compared to classical pathways, providing new insights into potential therapeutic approaches. The cGAS-STING pathway is distinct from traditional IRI-related pathways in that it is primarily activated by cytosolic double-stranded DNA (dsDNA), a signal of cellular damage. In the context of ischemic stroke, damaged cells release mitochondrial and nuclear DNA into the cytoplasm, where it serves as a DAMP that activates cGAS-STING signaling.

The cGAS-STING pathway has several unique advantages compared to classical inflammatory pathways. First, it serves as a direct sensor of cellular injury by recognizing cytosolic DNA, which is one of the earliest markers of cell damage. This allows cGAS-STING to respond swiftly to ischemia-induced cellular breakdown, potentially facilitating a timely immune response that is both localized and specific to the site of injury (76). Moreover, unlike pathways such as TLRs that rely on extracellular or endosomal signals, cGAS-STING operates directly within the cytoplasm, making it more responsive to intracellular disruptions associated with ischemia (77). Another notable feature of the cGAS-STING pathway is its dual role in both innate immune activation and the regulation of apoptosis. In the context of ischemic stroke, STING activation can lead to the production of cytokines that modulate both inflammatory and apoptotic pathways, impacting neurovascular integrity. This duality is advantageous because it may allow therapeutic targeting of both inflammatory and apoptotic processes, which are critical in limiting secondary brain injury post-stroke. Furthermore, research indicates that cGAS-STING activation can influence angiogenesis and neuroplasticity, suggesting a potential role in neural repair mechanisms (65, 78). Therefore, targeting cGAS-STING may not only mitigate initial injury but also promote recovery in the long term.

Despite these promising features, the role of cGAS-STING in IRI is not fully understood, and several limitations must be addressed in future research. One of the foremost challenges is the dual nature of the cGAS-STING pathway in inflammation and repair. While acute activation of cGAS-STING might help in initiating a protective immune response, prolonged or excessive activation could exacerbate inflammation and tissue damage, especially in the sensitive neural environment. Future studies should focus on delineating the temporal dynamics of cGAS-STING activation during IRI to determine the optimal time window for therapeutic intervention. Identifying when cGAS-STING signaling shifts from protective to detrimental could provide valuable insights for therapeutic development (79). Another limitation is the variability in cGAS-STING signaling responses across different cell types in the brain. While microglia, astrocytes, and neurons are known to exhibit distinct inflammatory responses, the specific roles of cGAS-STING in each cell type remain poorly defined. For example, STING activation in microglia may lead to a pro-inflammatory response, whereas in neurons, it could primarily influence apoptotic pathways (80). More research is needed to characterize cell-specific effects of cGAS-STING signaling, as this could enable more targeted therapies that mitigate inflammation without affecting neuronal survival.

## 7 Conclusion

The innate immune system is the body's first line of defense against pathogens, composed of various cellular and molecular mechanisms that can quickly recognize and respond to invading pathogens. The cGAS-STING pathway is one of the critical signaling pathways in the innate immune system, primarily involved in the recognition and response to intracellular DNA. Abnormal activation of the cGAS-STING pathway can lead to chronic inflammation and autoimmune responses, exacerbating damage and dysfunction in the body's own tissues. Current treatments for acute cerebral infarction mainly focus on reperfusion therapy during the acute phase to minimize brain tissue damage. However, the process of restoring blood flow can lead to further brain tissue damage, known as reperfusion injury. Reperfusion injury not only affects the treatment outcome but can also worsen neurological dysfunction.

The role of the cGAS-STING pathway in reperfusion in injury acute cerebral infarction has become a research hotspot. In mouse models of ischemia-reperfusion, the activation of the cGAS-STING pathway is closely associated with brain tissue damage and neurological dysfunction. Inhibiting cGAS or STING can reduce inflammation and oxidative stress, protect neurons, and improve outcomes. Additionally, cGAS or STING knockout mice exhibit significant neuroprotective effects in ischemia-reperfusion injury models, further confirming the critical role of this pathway in reperfusion injury. Therapeutic strategies such as cGAS inhibitors, STING inhibitors, and nano-drug delivery systems utilizing nanoparticles to deliver cGAS or STING inhibitors show great potential in mitigating reperfusion injury in acute cerebral infarction. Although most research to date has focused on animal models and *in vitro* experiments, more clinical studies are needed to verify the efficacy and safety of these approaches. With further research, therapeutic strategies targeting the cGAS-STING pathway are expected to provide new treatment options for patients with reperfusion injury in acute cerebral infarction.
